# Effect of Bi_2_O_3_ Particle Size on the Radiation-Shielding Performance of Free-Lead Epoxide Materials against Ionizing Radiation

**DOI:** 10.3390/polym16152125

**Published:** 2024-07-26

**Authors:** Ali Hedaya, Mohamed Elsafi, Wafa M. Al-Saleh, Ibrahim H. Saleh

**Affiliations:** 1 Environmental Studies Department, Institute of Graduate Studies and Research, Alexandria University, Alexandria 21511, Egypt; igsr.alihedaya@alexu.edu.eg (A.H.); igsr.ihindawy@alexu.edu.eg (I.H.S.); 2 Physics Department, Faculty of Science, Alexandria University, Alexandria 21511, Egypt; mohamedelsafi68@gmail.com; 3 College of Science and Health Professions, King Saud Bin Abdulaziz University for Health Sciences, Al-Ahsa 31982, Saudi Arabia; 4 King Abdullah International Medical Research Center, Al-Ahsa 31982, Saudi Arabia

**Keywords:** epoxy resin, Bi_2_O_3_-MPs, Bi_2_O_3_-NPs, attenuation coefficient, radiation-shielding efficiency, thermal analysis, morphological analysis

## Abstract

In this work, we studied the effect of bismuth oxide particle size and its attenuation capacity as a filler additive in epoxy resins. Six samples were prepared according to the amount of microparticles and nanoparticles in the sample and were coded as ERB-1, ERB-2, ERB-3, ERB-4, ERB-5, and ERB-6. One of the composite epoxies contained Bi_2_O_3_ microparticles at a 50:50 ratio (ERB-6) and was chosen as the control composite, and the number of microparticles (MPs) was gradually decreased and replaced by nanoparticles (NPs) to produce epoxy-containing Bi_2_O_3_ nanoparticles at a 50:50 ratio (ERB-1). The morphological and thermal characteristics of the studied composites were tested. The attenuation capability of the prepared composites, which is determined by the Bi_2_O_3_ particle size, was determined experimentally using a semiconductor detector, an HPGe-detector, and three different gamma-ray point sources (Am-241, Co-60, and Cs-137). The linear attenuation coefficient (LAC) of ERB-3, which contained 30% nanoparticles and 20% microparticles, had the highest value compared to the other composites at all the energies discussed, while the ERB-6 composite had the lowest value at all energies. The radiation-shielding efficiency (RSE) of the prepared samples was determined at all discussed energies; at 662 keV, the radiation-shielding efficiency values were 15.97%, 13.94%, and 12.55% for ERB-3, ERB-1, and ERB-6, respectively. The statistics also proved that the attenuation capacities of the samples containing a combination of nanoparticles and microparticles were much superior to those of the samples containing only microparticles or nanoparticles. A ranking of the samples based on their attenuation capacity is as follows: ERB-3 > ERB-4 > ERB-2 > ERB-1 > ERB-5 > ERB-6.

## 1. Introduction

In tandem with the overall growth in innovations in technology, there has been a notable spike in the development of radiation-using applications. Gamma rays and X-rays are forms of ionizing radiation that are frequently employed throughout a range of industries, such as the production of nuclear energy, imaging diagnostics, healthcare, agriculture, and academic research. Even though radiation offers many benefits, especially for medical professionals, it is well recognized that exposure to radiation for an extended period of time can have negative effects on both human health and the health of other living creatures [1,2,3,4].

To attenuate radiation, particles with a high atomic number and high density are required. Lead (Pb) and materials containing Pb have been preferred for ordinary radiation shielding due to their high density (11.29 g/cm^3^) and high atomic number (Z = 82). However, a significant issue is the toxicity of lead and Pb-based radiation-absorbing products for both people and the surrounding ecosystem, which necessitates the implementation of hygiene measures and waste disposal after usage [5,6,7]. Additionally, lead is extremely costly and cumbersome to wear for prolonged periods of time. Over the past decade, scientists have been searching for lightweight materials, flexible non-Pb composites that attenuate radiation, and other materials that are highly effective at attenuating radiation. To address these issues, different materials with a high Z have been evaluated [8,9].

Researchers are working extremely hard to create novel materials with effective radiation-shielding properties. Ceramics, alloys, glasses, building materials, and composites made of polymers are a few examples of these novel materials [10,11]. Novel shielding materials can also be created by altering the chemical structure of currently existing materials. Studies show that incorporating nano- or microparticles into particular materials can enhance their radiation-shielding ability [12,13,14].

Since many researchers have concentrated on fusing nanotechnologies with various materials like epoxy resin and other affordable materials to improve both the quality and the manufacturing of the mixture, it has recently been obvious that scientific research has made noteworthy progress in this discipline [15]. Materials that attenuate radiation commonly contain nanoparticle filler, which has a unique combination of qualities that enables it to absorb radiation efficiently. Recent research has shown that materials containing nanoparticle filler are more effective and efficient at shielding radiation than those containing bulk filler [16,17].

Epoxy resin, on the other hand, is a useful and important chemical in many fields due to its unique combination of properties. High durability and tenacity are two of the most important traits of epoxy resin. Additionally, it has superior resistance to chemicals, great thermal stability, and excellent electrical insulating properties. Epoxy resins are used in many different industries because of their unique blend of properties. Construction, engineering for civil purposes (to fix and strengthen concrete structures), dentistry, orthopedics, and the electrical and electronic sectors all commonly use epoxy resins. In the discipline of radiation attenuation, to make composites, epoxy resin can be utilized as a matrix that contains a combination of nanoparticles and microparticles to improve shielding properties [18,19,20].

Due to a unique set of properties that enables them to efficiently absorb radiation, nanoparticles made of bismuth oxide (Bi_2_O_3_) are extensively used in radiation shielding [21,22]. Recent research has shown that materials containing Bi_2_O_3_ nanoparticles have more proficient radiation-shielding properties than those containing Bi_2_O_3_ microparticles when taking into account efficacy and efficiency [23,24,25]. As a result of its high melting point and ability to withstand elevated temperatures without losing stability, Bi_2_O_3_ is suitable for use in a wide range of applications. Bi_2_O_3_ is nontoxic and relatively inexpensive compared to other substances in the industry that are used to attenuate radiation [26,27,28,29].

This work aims to develop new composites with nontoxic, nonhazardous ingredients and efficient radiation-shielding qualities for photon-shielding applications to overcome the negative impact of extended radiation exposure on the health of both humans and other living things.

## 2. Materials and Methods

Six solid epoxy samples were fabricated by grafting epoxy resin, which served as the matrix, with powdered bismuth oxide (Bi_2_O_3_), which is available in nano- and micro-sized (NP-MP) forms, as indicated by the ratios provided in Table 1, to create an enhanced radiation-shielding material.

### 2.1. Materials

#### 2.1.1. Epoxy Resin (ER)

Epoxy resin’s chemical composition is (C_21_H_25_ClO_5_). The ER is made up of two components, named basic epoxy substance and the hardener, and it is created by combining these two components. The hardener is pureed at a proportion of 5% of the basic epoxy substance added. Epoxy resin (ER) is utilized as a matrix viscous fluid substance due to it having a flexural strength of 55–70 N/mm^2^, tensile strength of 20–30 N/mm^2^, compressive strength of 90–100 N/mm^2^, and density of 1.05 g/cm^3^.

#### 2.1.2. Bismuth Oxide (Bi_2_O_3_)

**Figure 1:**
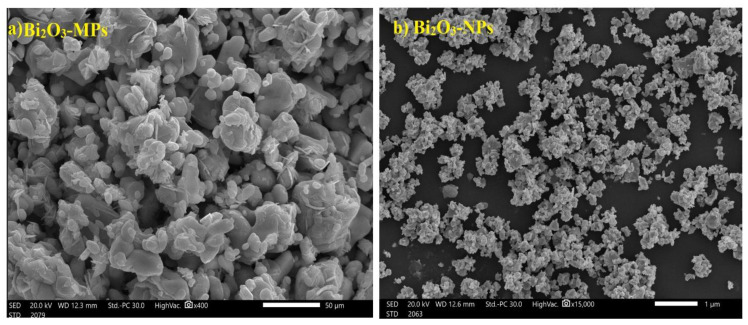
(**a**) TEM image of Bi_2_O_3_ microparticles and (**b**) SEM image of Bi_2_O_3_ nanoparticles.

Bi_2_O_3_ was selected as the filler because it possesses an intense absorption point (K-edge) at the energy of 88 keV. This point impacts both the absorption rate in this region and the dispersion of the particles within the mixture, and its high density. Using SEM, the average particle size of the Bi_2_O_3_ in nano- and micro-sized powder was determined, as shown in Figure 1.

### 2.2. Methods

To fabricate six epoxy resin composites by the ratios shown in Table 1, pure epoxy resin and Bi_2_O_3_ were added to the labeled glass beakers. After that, the composites were thoroughly agitated for 15 min until they had reached homogeneity. Then, they were inserted into mold boxes to avoid bubbles and kept in the lab for 36 h to dry at room temperature.

### 2.3. Shielding Measurements

**Figure 2:**
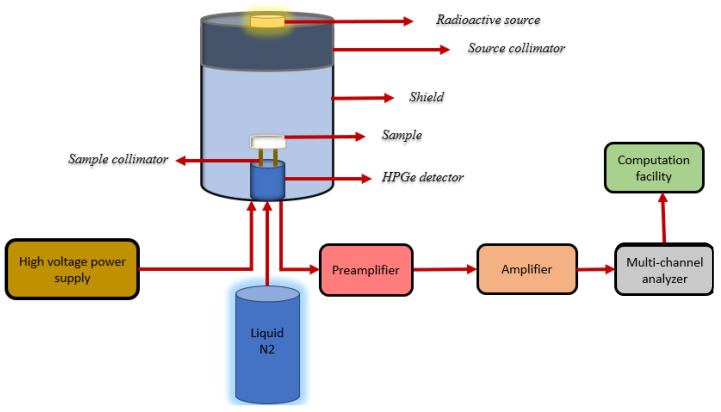
A diagram displaying the setup for radiation transmission measurements.

Utilizing a high purity germanium detector (HPGe-detector) manufactured by APTEC company in Canada. with various radioactive sources Cs-137 (decayed with one gamma energy 0.662 MeV), Co-60 (decayed with two gamma energies 1.173 and 1.333 MeV) and Am-241 (decayed with one energy 0.060 MeV), the photons attenuation performance of these samples was experimentally investigated as shown in Figure 2.

To confirm the accuracy of our work, we used the online software *Phy-X* to calculate the theoretical values of LAC at the four energy ranges for the pure micro powder sample (ERB-6). *Phy-X* software can predict the attenuation values for photon energies between 0.015 MeV and 100 MeV regarding any given material’s molecular or elemental structure [30]. The relative deviation between the experimental results and the theoretical (*Phy-X*) results is given by:

$$\mathrm{RD}\%=\frac{LAC\left(Phy-X\right)-LAC\left(Experimental\right)}{LAC\left(Experimental\right)}\times 100$$

To assess the component’s shielding performance, the linear attenuation coefficients (*LAC*) are experimentally determined, which are given by [31]:

(1) $$LAC=\frac{1}{x}ln\frac{I_{0}}{I}$$

where *I* is the gamma-ray intensity following absorber attenuation, *I*_0_ is the radiation intensity that the radioactive source emits when no absorber exists, and *x* is the thickness of the absorber.

The other shielding parameters were calculated as follows:

The required thickness of the absorbing material to bring down the incident rays on the material to one half of what it was initially (Half-value layer, *HVL*) [32]:

(2) $$HVL=\frac{ln(2)}{LAC}$$

The ratio of gamma-ray photon intensity transmits with and without an epoxy sample (Transmission factor, *TF*) [33]:

(3) $$TF=\frac{I}{I_{0}}$$

The mean distance that a photon takes to pass through a sample in the absence of any interactions (Mean free path, *MFP*) [32]:

(4) $$\mathrm{MFP}=\frac{1}{LAC}$$

The required thickness of the absorbing material to bring down the incident rays on the material to 10% of what it was initially (Tenth value layer, *TVL*) [32]:

(5) $$\mathrm{TVL}=\frac{ln(10)}{LAC}$$

The radiation-shielding effectiveness of epoxy composites, which assesses their attenuation abilities (Radiation-shielding efficiency, *RSE*) [34]:

(6) $$\mathrm{RSE}\%=[1-\frac{I}{I_{0}}]\times 100$$

## 3. Results

This section covers the effects of modifying polymeric epoxy resin with concentrations of Bi_2_O_3_ micro- and nanoparticles on enhancing the radiation-shielding efficiency of the produced composites.

**Figure 3:**
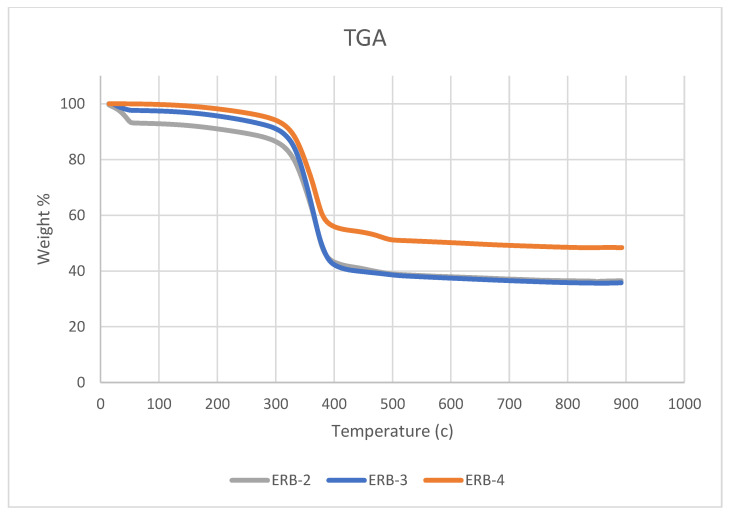
Weight % versus the Temperature of prepared composites.

It is known that high heat is generated with radiation. Therefore, the resilience of the fabricated samples under the effect of high temperatures must be taken into consideration. We elevated the temperature of the prepared samples with NP and MPs to 900 °C and monitored their behavior. Samples began to lose some of their mass gradually at approximately 200 °C, and then there was a surprising drop of mass after reaching approximately 300 °C and relative stability after reaching 420 °C, as shown in Figure 3. The ERB-4 sample with NP 20% showed more heat tolerance than the ERB-2 sample with NP 40% at temperatures below 400 °C. The ERB-4 sample kept 48.40% of the value of its original mass compared to 36.51% for ERB-2. Below 400 °C, the ERB-3 sample with NP 30% showed effective heat tolerance as the ERB-4 sample performed, but after 400 °C, the ERB-3 lost a lot of its mass until it reached 35.76% of its original mass value. The missing mass ratio is likely due to epoxy resin.

**Figure 4:**
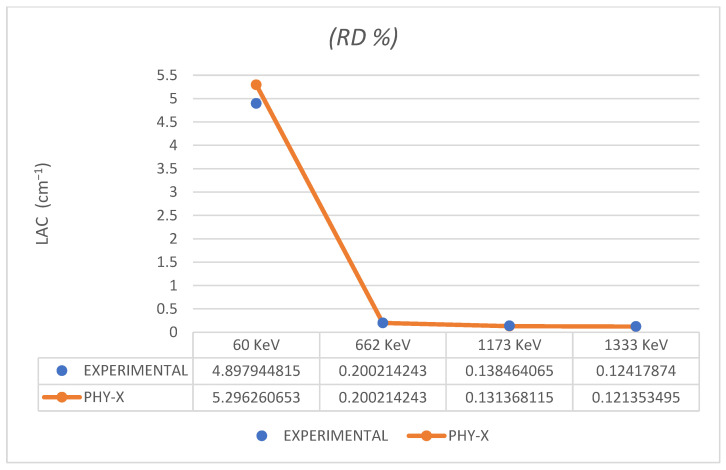
A diagram displaying the experimental results and the theoretical (Phy-X) results at four ranges of gamma energies. Am-241 (60 keV), Cs-137 (662 keV), Co-60 (1173 keV) and Co-60 (1333 keV).

For attenuation measurements, in order to determine the correctness of our samples, we had to compare the experimental value to the theoretical value we obtained using *Phy-X*, but it only matches the micro powder, and for that, we compared the experimental results of the LAC values of the ERB-6 to the *Phy-X* values at the four ranges of energies Am-241 (60 keV), Cs-137 (662 keV), Co-60 (1173 keV) and Co-60 (1333 keV), respectively. As illustrated in Figure 4, the theoretical output is expressed in a straight line, and the experimental output is expressed in dots. The relative difference values are acceptable, especially for the Cs-137 (662 keV), where it is 0.53%, and for the other energies, are 8.13%, −5.12%, and −2.28% for Am-241 (60 keV), Co-60 (1173 keV) and Co-60 (1333 keV), respectively.

To measure composite density-dependent, the Mass attenuation coefficient (MAC) was calculated (MAC=LAC/density of epoxide material) and reported in Table 2. The MAC was determined taking into account the calculation of errors.

**Figure 5:**
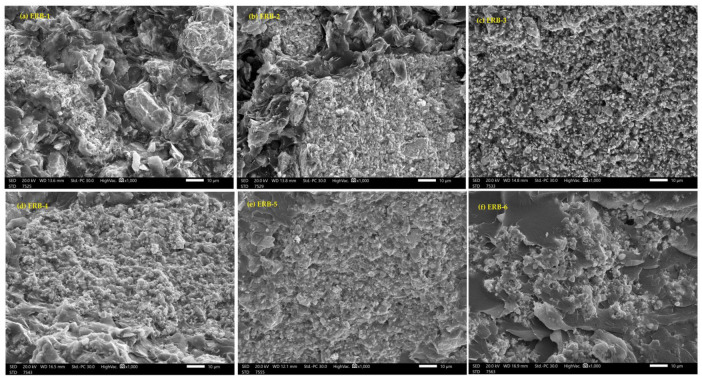
(**a**) SEM image of ERB-1, (**b**) SEM image of ERB-2, (**c**) SEM image of ERB-3, (**d**) SEM image of ERB-4, (**e**) SEM image of ERB-5, (**f**) SEM image of ERB-6.

Generally, the LAC values of these samples demonstrated that radiation attenuation depends on the particle size. For nano Bi_2_O_3_, LAC is higher than the micro Bi_2_O_3_. We found that the LAC for the ER sample with pure nanoparticle powder of Bi_2_O_3_ is higher than the ER sample with pure microparticle powder of Bi_2_O_3_ (ERB-1 > ERB-6). This observation is probably because of the particle distribution, which increased the LAC values for the NPs than the MPs, as shown in Figure 5. The small size of the nanoparticles offered even dispersion of the particles inside, enhanced the surface-to-mass ratio, and boosted the chance of gamma-ray interaction with the prepared ER samples. A similar observation was revealed by Cheewasukhanonta et al., who investigated how the attenuation performance was affected when nano and micro sizes of Bi_2_O_3_ were used in specific glass systems [30].

**Figure 6:**
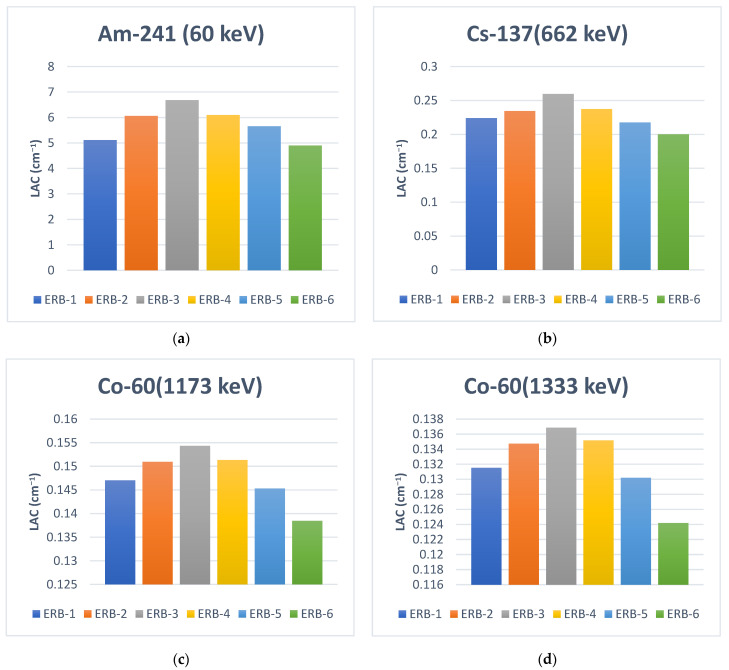
The linear attenuation coefficients (LAC) of the samples at four ranges of gamma energies. (**a**) Am-241 (60 keV), (**b**) Cs-137 (662 keV), (**c**) Co-60 (1173 keV) and (**d**) Co-60 (1333 keV).

In a specific look, we aimed to investigate the effect of particle size much more. As shown in Figure 6, the LACs of the nano Bi_2_O_3_ samples are higher than the micro Bi_2_O_3_ samples. For Am-241 (60 KeV) as an example, the LAC values are 6.6833, 5.1146, and 4.8979 (cm^−1^) for samples ERB-3, ERB-1, and ERB-6, respectively. This declares that the sample with 30% NPs—20% MPs has higher LAC than all other samples, including the pure NP sample (ERB-3 > ERB-4 > ERB-2 > ERB-1 > ERB-5 > ERB-6). After the process of pouring the samples containing Bi_2_O_3_ micro and nano-sized particles, we found that the sample was divided into layers, which increased scattering and chances of interaction. There is a very thin layer of Bi_2_O_3_ nanoparticles in front, followed by a larger layer of mixed and homogeneous Bi_2_O_3_ nanoparticles and microparticles, followed by a thin layer of Bi_2_O_3_ microparticles. This may be a result of the difference in density between nano Bi_2_O_3_ and micro Bi_2_O_3_. The LACs improvement is explained by the fact that the particles of varying sizes can interact with radiation in various energy ranges which enhances the total amount of attenuation of the sample. Furthermore, this rise in LAC could potentially be caused by the way the particles are distributed inside the composite sample. In other words, because of the abundance of nanoparticles within the microparticles, the composite sample can be more homogeneous than the sample containing nano Bi_2_O_3_. This opens up new horizons for thinking and novelty in this area to produce more future materials containing the nano-micro powder together in the same sample to determine the best ratio of them, leading to the best distribution of molecules in the sample and thus the best radiation attenuation.

**Figure 7:**
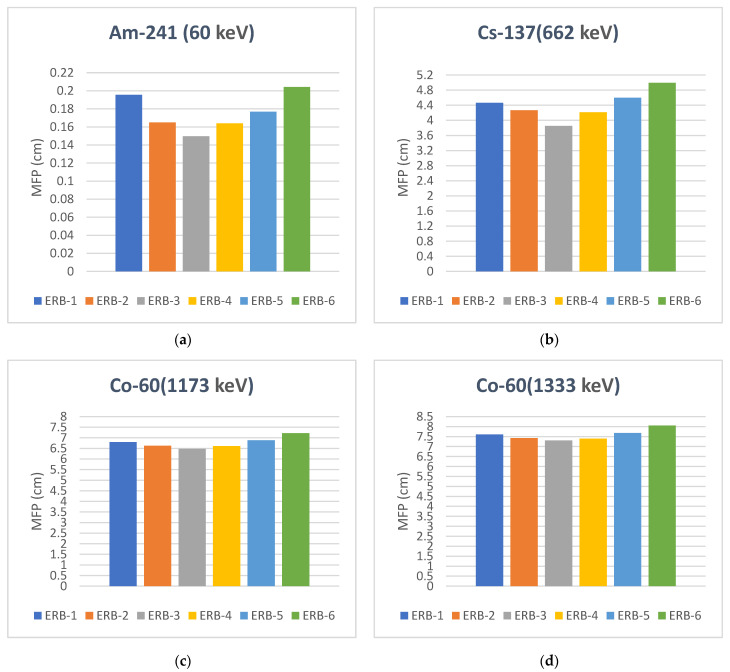
The mean free path (MFP) of the samples at four ranges of gamma energies. (**a**) Am-241 (60 keV), (**b**) Cs-137 (662 keV), (**c**) Co-60 (1173 keV) and (**d**) Co-60 (1333 keV).

To assess the attenuation performance of the shielding material according to the required shield thickness, it is vital to determine the MFP in the material. In Figure 7, the outcomes of the MFP calculations using the values of the LAC are shown. The mixed NP and MP samples show a greater ability to attenuate gamma radiation as it is associated with a shorter MFP. The higher the falling energy, the farther the gamma radiation’s ability to travel in the sample before losing its strength. From the results investigated, we have noticed that the mixed samples ERB-3, ERB-4, and ERB-2, respectively, have the shortest MFP over all other samples ERB-3 < ERB-4 < ERB-2 < ERB-1 < ERB-5 < ERB-6. When ERB-3 sample results were compared with the micro powder sample ERB-6 sample results, it was found that the MFP was reduced by 26.74%, 22.87%, 10.27%, and 9.26% at the four energies ranges Am-241 (60 keV), Cs-137 (662 keV), Co-60 (1173 keV) and Co-60 (1333 keV), respectively. When comparing the results of the ERB-3 sample with the results of the nanopowder ERB-2 sample, it was found that MFP was reduced by 23.48%, 13.72%, 4.7%, and 4.35% at the four energies ranges Am-241 (60 keV), Cs-137 (662 keV), Co-60 (1173 keV) and Co-60 (1333 keV), respectively. These findings make the fabricated samples have a wide range of potential applications, especially in sectors that need radiation protection equipment to be worn for long periods such as industrial and medical sectors. The results showed that it is achievable to utilize the present prepared composites as thinner shielding materials, which makes them lighter and more efficient.

**Figure 8:**
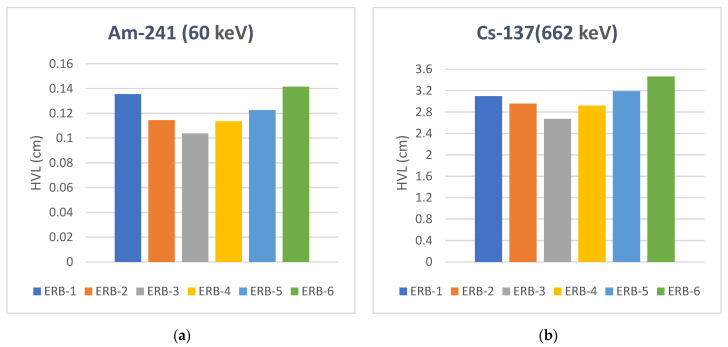

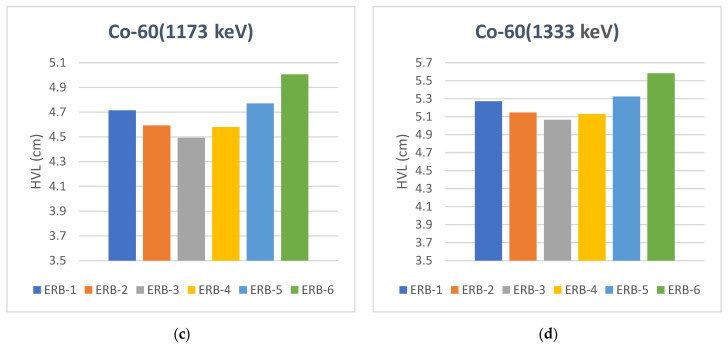
The half-value layer (HVL) of the samples at four ranges of gamma energies. (**a**) Am-241 (60 keV), (**b**) Cs-137 (662 keV), (**c**) Co-60 (1173 keV) and (**d**) Co-60 (1333 keV).

The half-value layer (HVL) reflects the economic dimension of the research and the success of the samples in reducing radiation using fewer raw materials. As shown in Figure 8., we observed that sample ERB-3 gave excellent results as it obtained the lowest estimate of the right thickness to reduce the intensity of the incident radiation to half its value, far from the sample ERB-1 of free NPs. At Am-241 (60 keV), for example, the HVL values are 0.1037, 0.1355, and 0.14152 cm for the samples ERB-3, ERB-1, and ERB-6, respectively. From here, we could say that the new fabricated sample ERB-3 has reduced the consumption of raw material by approximately 24% compared to the pure nano sample ERB-1 and by approximately 27% compared to the pure micro sample ERB-6. A higher photon energy corresponds with a lower ratio of the consumption of raw materials. For Cs-137 (662 keV), the ERB-3 sample of 2.7 cm thickness will bring down the incident rays on the material to one half of what it was. On the other hand, the sample ERB-6 must be 3.5 cm thick at least to reduce the intensity of the incident radiation to one half of its initial value. This is an important, exciting step forward in terms of reducing raw materials initiated, also reducing waste materials, and reducing the weight of shielding materials needed for protection in order to make it suitable to be worn for a prolonged period comfortably, and the reduction of the consumption raw material ratio becomes approximately 14% compared to the pure nano sample ERB-1 and approximately 23% for the pure micro sample ERB-6, This ratio reaches 5% for the other higher two ranges of energies. Hence, the HVL values results showed that the samples with different concentrations of NPs and MPs are smaller than the samples of pure MPs and pure NPs (ERB-3 < ERB-4 < ERB-2 < ERB-1 < ERB-5 < ERB-6), which declares the harmony between NPs and MPs in enhancing the radiation attenuation. The sample with high LAC is small in HVL value due to the fundamental formula stating an inverse relationship with the LAC.

**Figure 9:**
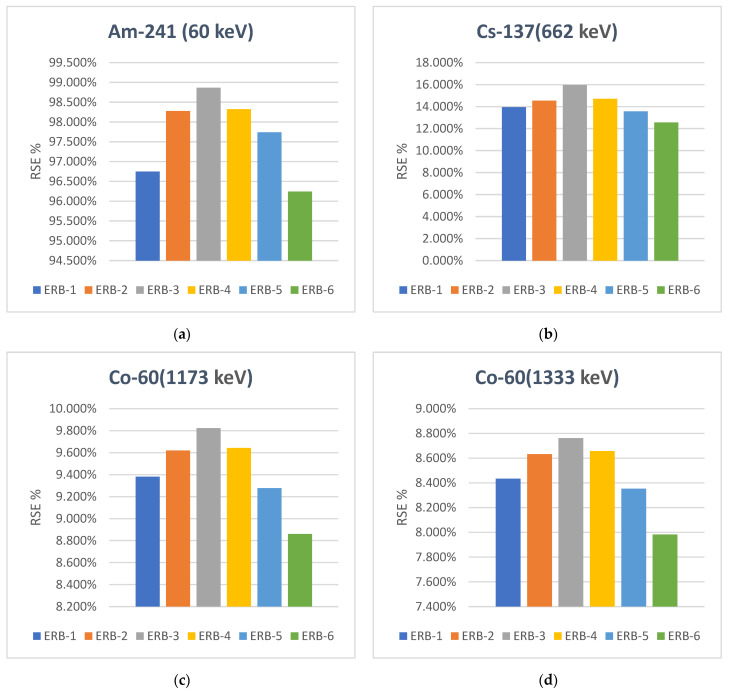
The radiation-shielding efficiency (RSE %) at four ranges of gamma energies. (**a**) Am-241 (60 keV), (**b**) Cs-137 (662 keV), (**c**) Co-60 (1173 keV) and (**d**) Co-60 (1333 keV).

The radiation-shielding efficiency (RSE%) is an additional important parameter. This provides clear details regarding the effectiveness of the particle size. Figure 9 shows the RSE statistics of the samples with an average thickness of 0.67 cm. In light of this figure, the sample that has nano and micro powder combined in a close ratio ERB-3 (20% MP—30% NP) has the highest RSE% among all samples. At Cs-137 (662 keV), for example, the radiation-shielding efficiency values are 15.97%, 13.94%, and 12.55% for the samples ERB-3, ERB-1, and ERB-6, respectively. The statistics also proved that the samples containing combined nano and microparticles are better than samples containing only nano or microparticles (ERB-3 > ERB-4 > ERB-2 > ERB-1 > ERB-5 > ERB-6). These findings emphasized the idea that nano and micro powder mixing homogenously is a very effective method of enhancing protection against ionizing radiation. The addition of nanoparticles to the epoxy sample results in an increase in the filler’s distribution, which increases the likelihood of interacting with incident gamma rays. The presence of microparticles in the sample gives homogenization to the sample with several protective layers which increase the number of refractions to which the falling photon goes through. Therefore, this attenuates the photon’s strength until it is absorbed, weakens, or becomes harmless, i.e., increasing the efficiency of the ionizing radiation protection process.

**Figure 10:**
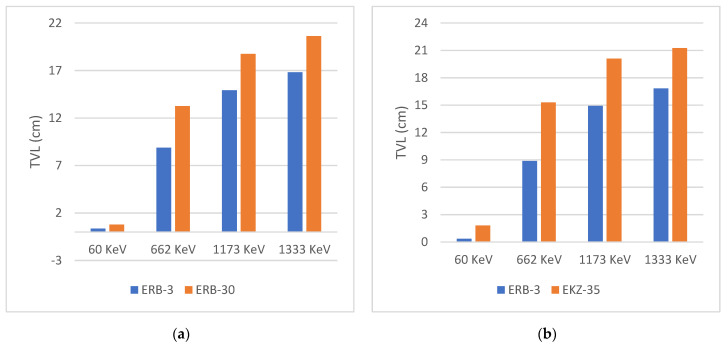
The tenth value layer for the fabricated samples at the four ranges of energies in comparison with other materials. (**a**) ERB-3 versus ERB-30 and (**b**) ERB-3 versus EKZ-35.

Comprehensively, we compared our TVL’s results to those of other materials from different research, such as EKZ-35 (40%Epoxy + 25% Kaolin Clay+ 35% ZnO Nanoparticles) [31] and ERB-30 (40%Epoxy + 30% Red clay + 30% Bi_2_O_3_ Nanoparticles) [32] at the four ranges of energies. Figure 10 illustrates that the TVL values for the ERB-3 sample are 0.3445, 8.8697, 14.9214, and 16.82547 cm at the four ranges of energies Am-241 (60 keV), Cs-137 (662 keV), Co-60 (1173 keV) and Co-60 (1333 keV) which are less values than the other chosen materials.

## 4. Conclusions

In this investigation, we manufactured novel radiation-shielding materials that are nontoxic, nonhazardous, affordable, and eco-friendly. The materials are made of epoxy resin as a matrix containing different amounts of Bi_2_O_3_ nano and microparticles. The results showed there was an excellent fit between the theoretical results of the LAC computed from the *Phy-X* software and the practical results for the sample ERB-6 (micro-composites). The linear attenuation coefficient of samples elevates significantly as the quantity of nano and microparticles increases. Moreover, the attenuation coefficients of the ERB-3 sample were significantly greater than the ERB-1 > ERB-6 sample due to the nanoparticles being more dispersed and spread in the sample, but the ERB-3 sample containing 30% nanoparticles and 20% microparticles may have greater homogeneity than the nano Bi_2_O_3_ sample due to the number of nanoparticles adjacent to the microparticles. The gamma radiation parameters HVL, TVL, RSE, and MFP were investigated as well, and the results demonstrated the superiority of sample ERB-3, which had the lowest value among all samples. At Am-241 (60 keV), the HVL values showed that the sample ERB-3 had reduced the consumption of raw material by approximately 24% and 27% compared to ERB-1 and ERB-6, respectively. Conclusively, the manufactured samples with a moderate quantity of Bi_2_O_3_ nano and microparticles have promising applications, particularly for industry and medical sectors. This work confirms that combining micro- and nanoparticles gives higher attenuation results than materials containing nanoparticles only, which reduces the cost of sample formation and is environmentally safe.

## Figures and Tables

**Table 1:** The chemical compositions and densities of the epoxide materials.

Sample Code	Percentage (wt.%)			Density (g cm^−3^)
	Epoxy Resin	Micro-Bi_2_O	Nano-Bi_2_O_3_	
ERB-1	50	0	50	2.013 ± 0.005
ERB-2	50	10	40	1.808 ± 0.010
ERB-3	50	20	30	2.254 ± 0.008
ERB-4	50	30	20	1.938 ± 0.005
ERB-5	50	40	10	2.130 ± 0.009
ERB-6	50	50	0	1.836 ± 0.007

**Table 2:** The MAC of prepared epoxied materials at different energies.

Composite Name	MAC, g cm^−2^			
	60 keV	662 keV	1173 keV	1333 keV
ERB-1	2.5408 ± 0.0016	0.1113 ± 0.0010	0.0730 ± 0.0015	0.0653 ± 0.0018
ERB-2	3.3514 ± 0.0010	0.1296 ± 0.0023	0.0835 ± 0.0013	0.0745 ± 0.0009
ERB-3	2.9651 ± 0.0009	0.1152 ± 0.0009	0.0685 ± 0.0021	0.0607 ± 0.0015
ERB-4	3.1480 ± 0.0014	0.1225 ± 0.0018	0.0781 ± 0.0018	0.0697 ± 0.0011
ERB-5	2.6560 ± 0.0019	0.1021 ± 0.0013	0.0682 ± 0.0019	0.0611 ± 0.0017
ERB-6	2.6677 ± 0.0021	0.1090 ± 0.0020	0.0754 ± 0.0011	0.0676 ± 0.0021

## Data Availability

The original contributions presented in the study are included in the article. Further inquiries can be directed to the corresponding author.
